# Low-Dose Radiation to COVID-19 Patients to Ease the Disease Course and Reduce the Need of Intensive Care

**DOI:** 10.2967/jnumed.120.251892

**Published:** 2020-12

**Authors:** Poul Flemming Høilund-Carlsen, Poul-Erik Braad, Oke Gerke, Kasper Karmark Iversen, Werner Vach

**Affiliations:** * Odense University Hospital Sdr. Boulevard 29 Odense C, 5000, Denmark E-mail: pfhc@rsyd.dk

**TO THE EDITOR:** Several recent studies relate to the use of PET/CT to assess pulmonary inflammation and precautions in general when COVID-19 patients are in need of nuclear medicine investigations (1,2). We would like to call attention to another, rather unnoticed but potentially promising, therapeutic application of what we have previously termed “the good rays” (3), that is, the treatment of COVID-19–infected patients with low-dose radiation (LDR), which we believe will not only benefit these patients significantly, but also substantially improve the therapeutic capacity of the health-care system.

An increasing body of evidence suggests that LDR has beneficial effects on the body’s DNA repair and immune systems (4). These properties may render LDR attractive for the treatment of COVID-19 patients. LDR has no short-time side effects and is widely available, easy to administer, and inexpensive. This is in contrast to several of the currently discussed alternatives for treatment of COVID-19, many of which are under investigation in randomized controlled trials (RCTs), a circumstance that may be of particular interest in countries with limited resources and insufficient access to health care.

A hundred years of observations from studies of intended and unintended irradiation of animals and humans have consistently and almost unanimously demonstrated that effective doses to the body of up to 200 or 250 mSv are not only harmless, but also beneficial in being associated with decreased rates of cancer and increased longevity (3). These many observations have not sufficed to make authorities slacken current rules of radiation protection that build on (unscientific) linear extrapolation from tissue damage observed with high radiation dose to the theoretic damage that the model claims is caused by LDR. This *linear no-threshold (LNT)* model has never been validated in the LDR range. In contrast, it has consistently been contradicted by a multitude of observations (3).

Time has come to make up for this mistake and exploit LDR to fight COVID-19—as a simple and stand-alone therapeutic procedure or as an adjunct to other forms of therapy being tested. Historically, x-rays have been used during the first half of the 20th century to successfully treat bacterial, sulfanilamide-resistant, interstitial, and atypical (including viral) pneumonia, a single treatment with low doses of x-ray quickly relieving respiratory distress and markedly reducing the risk of mortality, especially when given early in the disease course (5). With regard to the biologic effects of ionizing radiation, multiple systems are involved. However, their net effect at doses of 100–300 mSv are antiinflammatory actions minimizing toxicity, whereas increasing proinflammatory effects are observed at larger doses (6); that is, a biphasic response consistent with Edward Calabrese’s demonstration from thousands of biologic systems that this is nature’s “law” rather than a straight association as the one postulated by the LNT model (7).

We expect LDR irradiation to prevent or significantly relieve the severe pneumonia, which some COVID-19 patients develop and which may cause their death due to multiorgan failure because of insufficient oxygen supply. Quantifying the effect of LDR to be expected in COVID-19 patients is challenging due to lack of experience in comparable populations. However, the available evidence on the possible effect of LDR on short-term and long-term outcomes from clinical and animal studies suggests that substantial effects are not unlikely (5,8,9). A conservative guess would be that LDR will shorten the duration of the disease and reduce the number of patients in need of intensive care by one third and shorten the intensive care unit stay by at least 20%, that is, something that will have a substantial benefit on health system capacity (10). Moreover, we can build on a long-term experience with LDR as pretreatment in patients with multiple myelomas and certain other cancers (11,12), which allows for monitoring and controlling the potential risk associated with the frequent exposure of the health-care staff involved in administering LDR to COVID-19 patients.

It is probably rather safe to use LDR as an adjunct to other pharmacologic interventions because a direct interference with the pathways affected by pharmacologic interventions is unlikely—in contrast to combining different pharmacologic interventions. In this way, LDR also offers an opportunity to overcome the limitations implied by the limited effectiveness of any single intervention. For the same reasons, LDR can be tested in RCTs alongside a pharmacologic intervention in a 2 × 2 factorial design, avoiding excluding participants from benefiting from other promising options and offering three fourths of all participants an active treatment. An RCT on effects of LDR in COVID-19 patients requires little preparation except for ethical approval and access to instruments capable of providing LDR such as planar x-ray or CT equipment and, hence, is fast to implement. The beneficial effect of LDR can be an advantage at all stages of disease, thus offering a wide spectrum for its application. It might be applied simultaneously to other interventions or may fill the time gap between them. To what extent ^18^F-FDG PET/CT can come into use for examination of the lungs of these patients and in particular for monitoring effects of therapy is another, but also very interesting, question. We are very willing to collaborate with researchers who are interested in adding LDR as an adjunct to ongoing or coming RCTs on COVID-19 therapeutic options.
